# The Influence of Materials on the Mechanical Properties of Ultra-High-Performance Concrete (UHPC): A Literature Review

**DOI:** 10.3390/ma17081801

**Published:** 2024-04-14

**Authors:** Mariana Lage da Silva, Lisiane Pereira Prado, Emerson Felipe Félix, Alex Micael Dantas de Sousa, Davi Peretta Aquino

**Affiliations:** 1 Department of Civil Engineering, School of Science and Engineering, São Paulo State University (UNESP), Guaratinguetá 12516-410, SP, Brazil; mariana.lage@unesp.br (M.L.d.S.); emerson.felix@unesp.br (E.F.F.); peretta.aquino@unesp.br (D.P.A.); 2 Department of Civil Engineering, School of Engineering, São Paulo State University (UNESP), Ilha Solteira 15385-000, SP, Brazil; alex.dantas@unesp.br

**Keywords:** ultra-high-performance concrete, materials, mechanical properties, statistical analyses

## Abstract

Ultra-high-performance concrete (UHPC) is a cementitious composite combining high-strength concrete matrix and fiber reinforcement. Standing out for its excellent mechanical properties and durability, this material has been widely recognized as a viable choice for highly complex engineering projects. This paper proposes (i) the review of the influence exerted by the constituent materials on the mechanical properties of compressive strength, flexural tensile strength, and elastic modulus of UHPC and (ii) the determination of optimal quantities of the constituent materials based on simplified statistical analyses of the developed database. The data search was restricted to papers that produced UHPC with straight steel fibers at a content of 2% by volume. UHPC mixture models were proposed based on graphical analyses of the relationship of constituent materials versus mechanical properties, aiming to optimize the material’s performance for each mechanical property. The results proved to be in accordance with the specifications present in the literature, characterized by high cement consumption, significant presence of fine materials, and low water-to-binder ratio. The divergences identified between the mixtures reflect how the constituent materials uniquely impact each mechanical property of the concrete. In general, fine materials were shown to play a significant role in increasing the compressive strength and flexural tensile strength of UHPC, while water and superplasticizers stood out for their influence on the material’s workability.

## 1. Introduction

Ultra-high-performance concrete (UHPC), developed to overcome the limitations of conventional concrete, has been increasingly used in highly complex engineering projects that require better mechanical and durability properties. The construction of prestressed bridges with reduced cross-section and large spans is among these projects [1,2,3,4]. It has also been applied as a filling material in the connection region of precast concrete structures at the construction site, playing a significant role in achieving the monolithic behavior of the elements [5]. Furthermore, existing technologies in precast concrete factories have facilitated UHPC production and application as a filling material in connections subject to high stress [6].

High mechanical strength, durability, ductility, and ability to adhere to other concretes differentiate UHPC from conventional concrete. Ultra-high-performance concrete is an excellent material for connecting due to its strength, adhesion to hardened concrete, and small particle size that can fill congested regions [7].

These characteristics are achieved by the high amount of cement, mineral additions (silica fume, quartz powder or quartz filler, and fly ash), fine aggregate, and low water-to-binder ratio, increasing the consumption of superplasticizers. Thus, it maximizes the relative density and reduces capillary pore size and volume, leading to a more durable microstructure [8,9,10,11,12,13,14,15].

Ultra-high-performance concrete is distinct from conventional concrete due to the inclusion of ultrafine granular components with a maximum diameter of less than 2 mm. These materials include Portland cement, fine sand, silica fume, and quartz powder [7,16]. If the concrete contains granular materials with particles larger than 2 mm, such as coarse aggregate, it can cause heterogeneity. This can lead to cracks forming in the transition zone at the paste/aggregate interface, reducing the material’s compressive strength [17].

Incorporating Supplementary Cementitious Materials (SCMs) in the UHPC mixture can improve the sustainability of the concrete without compromising its performance. Materials such as quartz powder and silica fume, which were not extensively used in conventional concrete, now play a crucial role in enhancing the mechanical and durability properties of UHPC. Quartz powder and silica fume stand out among the mineral additions used in mixing ultra-high-performance concrete as fundamental components for gaining strength and fluidity. Quartz powder addition aims to optimize the UHPC particle size, providing a uniform distribution between the grains of fine sand and cement [16]. Furthermore, studies have indicated the possibility of replacing up to 30% of the cement volume with quartz powder without reducing compressive strength [18]. In turn, silica fume incorporation promotes the formation of a dense and low-porosity cementitious matrix, allowing better packing of particles and increasing the plasticizing effect, filling the empty spaces between the cement and the quartz powder that would be occupied by water particles, allowing them to be redistributed throughout the concrete structure, and improving fluidity [19].

As a result of the substantial presence of fine materials in the concrete composition, the enhancement of its fluidity is attained through the incorporation of superplasticizer additives. These additives are frequently integrated into the mixture to reduce the water-to-binder ratio to values ranging from 0.10 to 0.20 by mass. This meticulous adjustment aims to optimize the mechanical properties of concrete [20].

This set of specifications results in a cementitious composite whose compressive strength is higher than 130 MPa [16,21,22,23,24,25,26,27,28,29,30,31], the flexural strength is higher than 20 MPa [23,24,25,26,27,28,30,31,32,33,34], and the elastic modulus is higher than 30 GPa [16,21,24,29,32,34,35,36,37,38].

The high gain in strength in ultra-high-performance concrete causes the material to exhibit stiff behavior, characterized by small strain relative to the achieved level of strength. Steel fibers are commonly incorporated into the mixture to prevent the occurrence of brittle failure and promote higher strain capacity [7]. Fibers act as stress transfer bridges, distributing stress in the cracks and preventing their unstable propagation, giving the concrete a higher capacity to withstand stress and strain after reaching strength, and preventing brittle rupture [39,40]. However, the peak resistance and corresponding strain up to the formation of the first crack do not undergo significant changes compared to conventional concrete [41].

Despite being considered one of the most promising materials in construction, the feasibility of ultra-high-performance concrete is compromised due to its high production cost and the associated environmental impact. Therefore, in pursuit of a more affordable and sustainable material, researchers have devoted efforts to developing alternative UHPC formulations using locally available materials [42,43,44]. One of the most promising alternatives is partially replacing silica fume with rice husk ash. In addition to reducing the production cost of UHPC, the incorporation of rice husk ash enhances the hydration of cement and provides the concrete with compressive strength values exceeding 150 MPa [45,46]. Another effective option is substituting a portion of fine sand and cement with recycled waste glass, which has reduced concrete permeability and increased workability [47]. In addition to the adjustments made to the fine materials, another notable aspect in UHPC improvement studies is integrating a hybrid fiber system into the concrete composition, combining steel fibers of different dimensions and geometries. Studies indicate that macrofibers perform better in post-cracking strength, deformation capacity, and multiple cracking behaviors. At the same time, microfibers enhance tensile strength and reduce the occurrence of microcracks [48].

Other studies have also investigated the optimal design of the mixture based on advanced statistical analyses, for instance, considering the interaction of the different raw materials on the mechanical properties of the UHPC [49,50]. Machine learning methods can be used to correlate mechanical qualities or durability with material proportions to achieve the optimal concrete mix, particularly for UHPC [51,52,53,54,55]. In practice, this allows a comprehensive understanding of the effect of various materials on the mix. However, the requirement for a larger database and a more complex set of operations can hinder the widespread adoption of these methods.

This research was aimed at evaluating how each individual material affects the mechanical properties of UHPC. It proposed theoretical mixture models to assist in creating compositions that better match the actual material conditions. The objective is to enhance the material’s compressive strength, flexural tensile strength, and elastic modulus to achieve peak performance. Finally, we intend to verify the consistency of the theoretical results by comparing them with experimental studies available in the literature, identifying possible divergences that could guide future studies. This paper addresses a knowledge gap in the field of UHPC by suggesting a reference mixture for ultra-high-performance concrete based on a review of numerous UHPC mixtures, making a vital contribution to the research community. Developing a UHPC mixture with the desired mechanical properties is difficult, and determining a reference mixture can minimize the amount of laboratory testing required. The proposed method has a limitation in that it only considers the effect of individual materials and does not account for how these materials interact to affect the material’s mechanical properties.

## 2. Methodology

Electronic platforms commonly used in academic research, such as Scopus and Science Direct, both in national and international literature, were searched to develop this bibliographic review. In addition, university repositories were also used.

The selection of research sources was guided by specific keywords related to the review topic, namely:

- Ultra-high-performance concrete (UHPC);
- Steel fiber;
- Compressive strength;
- Flexural tensile strength;
- Elastic modulus;
- Proportion of materials.

Inclusion and exclusion criteria were established to provide higher control over the results and ensure the selectivity of data relevant to the research (Table 1).

Twenty-one studies were selected to integrate the literature review, from which information was extracted about the constituent materials of UHPC and experimental results of mechanical properties. The constituent materials in the studies were grouped into cement, fine aggregate, silica, quartz powder, mineral additions from waste (fly ashes and GGSB), water, and superplasticizer. The information collected corresponded to the mechanical properties comprising compressive strength, flexural tensile strength, and elastic modulus (Table 2).

**Figure 1:**
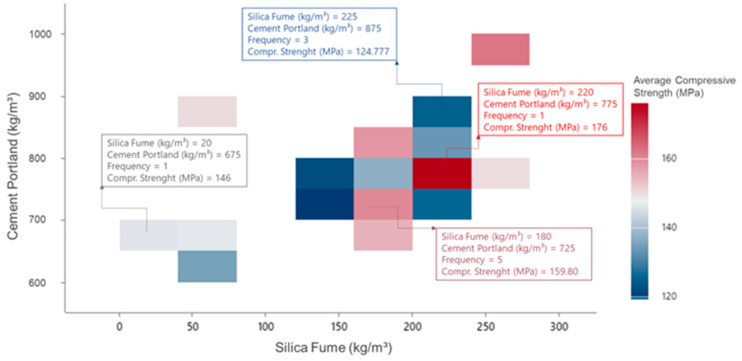
Relationship between cement and silica fume consumptions with the compressive strength.

Information regarding the consumption of constituent materials and mechanical properties of UHPC was extracted from the dataset defined with the studies presented in Table 2, which were subsequently subjected to three analyses. Scatterplots representing the relationships between each component’s consumption and the ultra-high-performance concrete mechanical properties were created in the first analysis. Boxplots were made for the second analysis, offering a detailed view of the average trends and variations in the consumption of UHPC constituent materials in each mechanical property. The third analysis was developed in the software MINITAB^®^ 21.2. It consisted of creating block scatterplots that related the proportions of two constituent materials with a mechanical property of UHPC, as exemplified in Figure 1.

**Figure 2:**
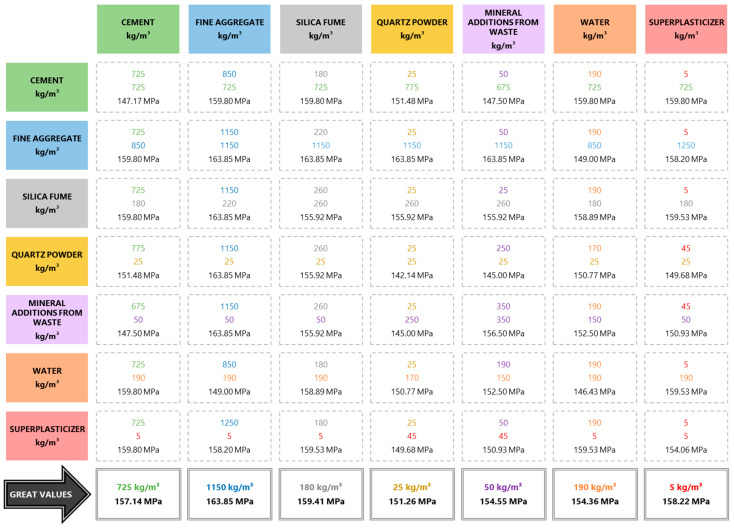
Relationship between constituent materials and compressive strength.

With the block scatterplots, it was possible to evaluate the combined relationship of two materials with the mechanical properties and, using the mode, to infer which were the combined proportions most used in the literature to generate UHPC. Once the most used proportions had been defined, it was possible to infer the average value of the mechanical properties of the concretes generated with these proportions and then define the ideal theoretical UHPC proportioning mixture with the most frequently used material consumptions (Figure 2). This procedure was adopted for analyses related to compressive strength, flexural strength, and modulus of elasticity properties.

In Figure 1, we can see the analysis conducted to determine the most commonly used amounts of silica fume and Portland cement for making concrete. It was found that 180 kg/m^3^ of silica fume and 725 kg/m^3^ of cement was the most commonly used consumption combination (5 concretes were produced with these values), as shown in the reddest block scatter. This combination resulted in ultra-high-performance concrete with an average compressive strength of 159.80 MPa.

Figure 2 shows the most used consumptions and their association with the concrete compressive strength. Furthermore, to generate the ideal theoretical proportion, considering the database, the most frequent value of each material was determined, where the results are presented in the last line of the block of information shown in Figure 2. Thus, it was observed that the most frequent consumptions are 725 kg/m^3^ of cement, 1150 kg/m^3^ of fine aggregate, 180 kg/m^3^ of silica fume, 25 kg/m^3^ of quartz powder, 50 kg/m^3^ of mineral additions from waste, 190 kg/m^3^ of water, and 5 kg/m^3^ of superplasticizers. With these theoretical UHPC mix design proportions, the compressive strength would fluctuate between 151.26 MPa and 163.85 MPa.

This work’s established approach provides a representative mechanical property value for the mixture as a whole, considering each component’s unique properties.

## 3. Results and Discussion

Section 3.1, Section 3.2, Section 3.3, Section 3.4, Section 3.5, Section 3.6 and Section 3.7 describe the results and discussions regarding the effect of different constituent materials on the mechanical properties of the UHPC mixture. Section 3.8 proposes a reference mixture for UHPC. In Section 3.9, the reference mixture proportions are compared to those studied by researchers in terms of mechanical properties. Lastly, Section 3.10 presents a study on the relationship between UHPC’s compressive strength and other properties such as modulus of elasticity and flexure tensile strength.

### 3.1. Influence of Cement on the UHPC Mixture

**Figure 3:**
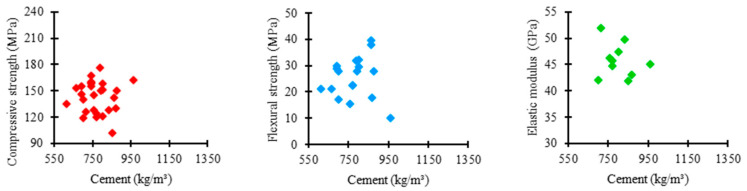
Proportion of cement × mechanical properties.

Figure 3 shows the relationship between the amount of cement used in the UHPC mixtures in the consulted studies and the respective obtained mechanical properties. Cement consumption tends to converge between 550 and 950 kg/m^3^, as presented by most authors. In this range, the compressive strength varied between 120 and 180 MPa, flexural strength between 10 and 40 MPa, and elastic modulus between 40 and 50 GPa.

**Figure 4:**
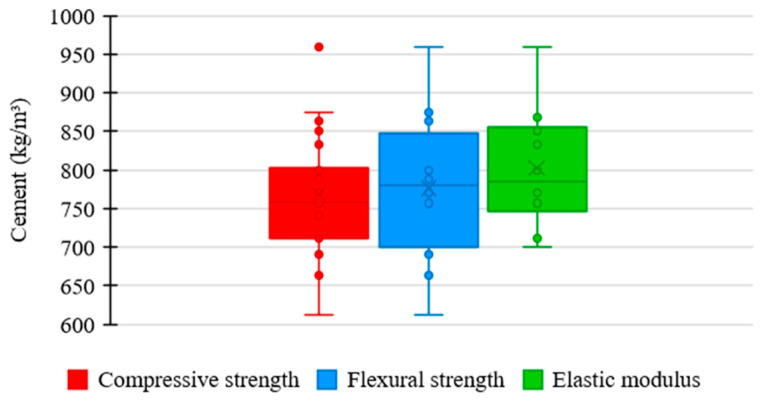
Tendency of cement consumption distribution × mechanical properties of UHPC.

Figure 4 displays the distribution trends in cement consumption in the UHPC mixtures of the data regarding compressive strength, flexural strength, and elastic modulus.

Regarding compressive strength, the mean trend in the amount of cement among the concrete mixtures was 757 kg/m^3^, the lowest value recorded among the analyzed properties. It shows that cement content significantly influences the compressive strength of UHPC, not requiring excessive consumption of the material to achieve satisfactory results. Moreover, the reduced box width stands out, indicating higher uniformity between the amounts of cement used in the mixtures, which suggests strict standardization relative to the proportion of this material.

Regarding flexural strength, the mean trend in cement consumption among concrete mixtures was 780 kg/m^3^, an intermediate value between the studied properties. It reveals that flexural strength is not influenced as significantly by cement content as compressive strength. Also, the median range displayed by the box suggests a moderate uniformity between the proportions of cement used in the mixtures, demonstrating a slightly rigorous standardization relative to the consumption of this component.

Regarding elastic modulus, the mean trend in the proportion of cement among the concrete mixtures was 786 kg/m^3^, the highest value recorded among the examined properties. It establishes that cement content does not significantly influence the elastic modulus of UHPC. The high box range also stands out, representing a higher dispersion between quantities of cement incorporated in the mixtures, which indicates a more flexible standardization relative to the proportion of this constituent.

### 3.2. Influence of Fine Aggregate on the UHPC Mixture

**Figure 5:**
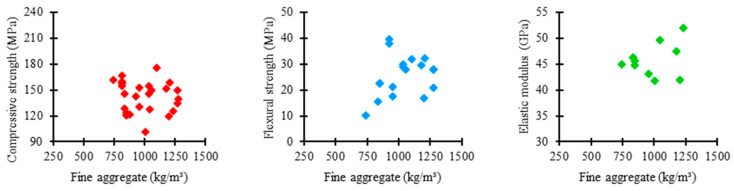
Proportion of fine aggregate × mechanical properties.

Figure 5 shows the relationship between the amount of fine aggregate used in the UHPC mixtures of the examined studies and the corresponding mechanical properties. As presented by most authors, the consumption of fine aggregate tends to converge between 750 and 1250 kg/m^3^. In this range, the compressive strength varied between 120 and 180 MPa, flexural strength between 10 and 40 MPa, and elastic modulus between 40 and 50 GPa.

**Figure 6:**
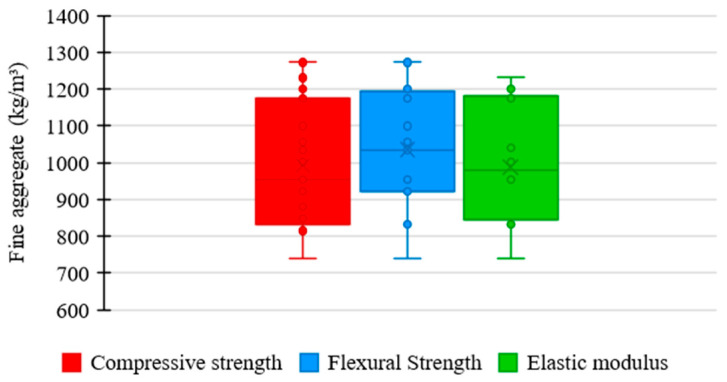
Tendency of fine aggregate consumption distribution × mechanical properties of UHPC.

Figure 6 illustrates the distribution trends of fine aggregate consumption in UHPC mixtures in the explored data with respect to compressive strength, flexural strength, and elastic modulus.

Regarding compressive strength, the mean trend in the fine aggregate consumption among the concrete mixtures was 955 kg/m^3^, the lowest value recorded among the analyzed properties. It shows that the fine aggregate content significantly influences the flexure of UHPC, not requiring an exorbitant consumption of material for a satisfactory performance to be achieved. However, a high box amplitude is noted, which indicates higher variability between the proportions of fine aggregate used in the mixtures, suggesting a more flexible standardization relative to the quantity of this component.

Regarding flexural strength, the mean trend in fine aggregate consumption among concrete mixtures was 1035 kg/m^3^, the highest value recorded among the studied properties. It shows that the fine aggregate content has no relevant influence on the flexural strength of UHPC, with a considerable proportion of material being required to achieve satisfactory results. Despite this, the box has a reduced range, revealing a high uniformity between the amounts of fine aggregate used in the mixtures, which represents a rigorous standardization relative to the consumption of this constituent.

The mean trend in the proportion of fine aggregate among the concrete mixtures was 978 kg/m^3^ for the elastic modulus, an intermediate value recorded between the examined properties. It demonstrates that the influence exerted by fine aggregate on the elastic modulus of UHPC is not as noticeable as on compressive strength, requiring a high amount of material to provide satisfactory performance. However, a medium range is noted for the box, which suggests a moderate uniformity between the proportions of fine aggregate incorporated in the mixtures, establishing a slightly strict standardization relative to the consumption of this material.

### 3.3. Influence of Silica Fume on the UHPC Mixture

**Figure 7:**
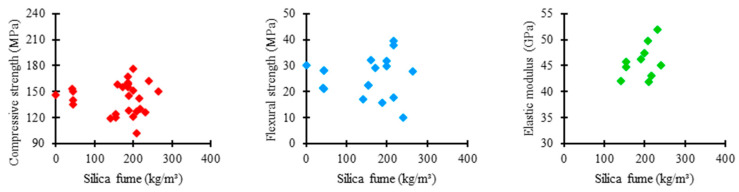
Proportion of silica fume × mechanical properties.

Figure 7 shows the relationship between the amount of silica fume used in the UHPC mixtures of the analyzed studies and the respective mechanical properties. As presented by most authors, silica fume consumption tends to converge between 100 and 300 kg/m^3^. In this range, the compressive strength varied between 120 and 180 MPa, flexural strength between 10 and 40 MPa, and elastic modulus between 40 and 50 GPa.

**Figure 8:**
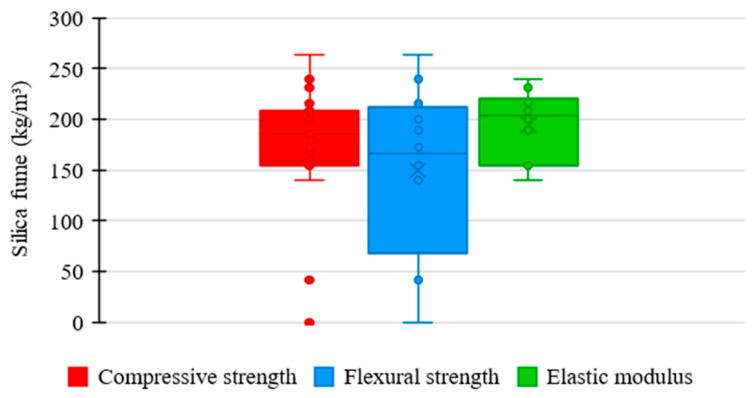
Trends of silica fume consumption and the influence on mechanical properties of UHPC.

Figure 8 shows the distribution trends of silica fume consumption in UHPC mixtures from the consulted studies regarding compressive strength, flexural strength, and elastic modulus.

Regarding compressive strength, the mean trend in the amount of silica fume among the concrete mixtures was 185 kg/m^3^, an intermediate value recorded between the analyzed properties. It suggests a significant moderate influence of the silica fume content on the compressive strength of UHPC, requiring a higher consumption of material to obtain satisfactory results. Furthermore, the box shows a low range, representing considerable uniformity between the amounts of silica fume used in the mixtures, which indicates a rigorous standardization relative to the proportion of this material.

Regarding flexural strength, the mean trend in silica fume consumption among concrete mixtures was 166 kg/m^3^, the lowest value recorded among the studied properties. It proposes that the silica content significantly influences the flexural strength of UHPC, not requiring an exorbitant amount of material to achieve satisfactory performance. However, a relatively high range of the box is observed, which establishes higher variability between the proportions of silica fume used in the mixtures, revealing a more flexible standardization relative to the consumption of this component.

Regarding elastic modulus, the mean trend in the proportion of silica fume among the concrete mixtures was 204 kg/m^3^, the highest value recorded among the examined properties. It shows a low influence of silica fume on the elastic modulus of UHPC, requiring a high consumption of material to obtain satisfactory results. Another important point is the box with a medium range, demonstrating a moderate uniformity between the proportions of silica fume incorporated in the mixtures, which indicates a slightly rigorous standardization relative to the amount of this constituent in the different studies.

### 3.4. Influence of Quartz Powder on the UHPC Mixture

**Figure 9:**
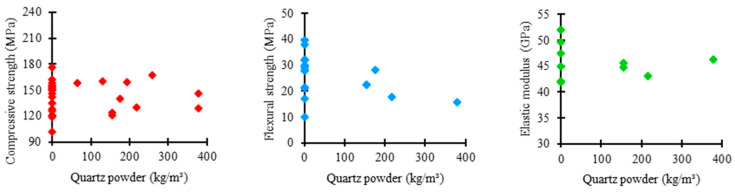
Proportion of quartz powder × mechanical properties.

Figure 9 shows the relationship between the amount of quartz powder used in the UHPC mixtures in the examined studies and the corresponding mechanical properties. As presented by most authors, quartz powder was not incorporated into concrete mixtures. However, additions in research that used the material occurred between 100 and 300 kg/m^3^. In this range, the compressive strength ranged between 120 and 180 MPa, flexural strength between 10 and 30 MPa, and elastic modulus between 40 and 45 GPa.

**Figure 10:**
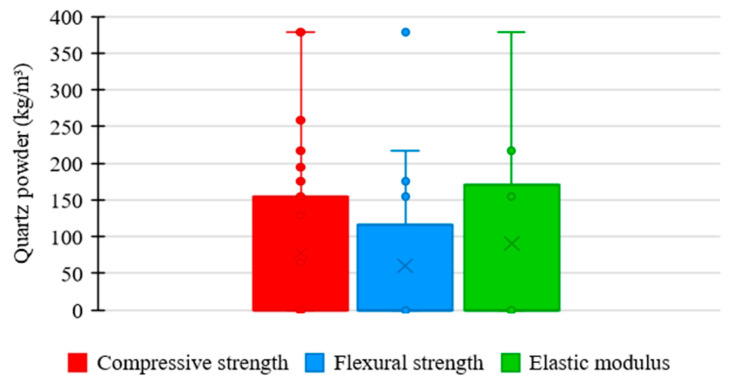
Trends of quartz powder consumption distribution × mechanical properties of UHPC.

Figure 10 displays the distribution trends in quartz powder consumption in the UHPC mixtures based on the studied data regarding compressive strength, flexural strength, and elastic modulus.

The mean trend in quartz powder consumption among the ultra-high-performance concrete mixtures was 0 kg/m^3^ for the three analyzed mechanical properties. This shows that quartz powder is not commonly investigated due to its influences on UHPC properties.

However, the box range for compressive strength appears to be medium relative to the others, indicating a balanced uniformity between the quantities of quartz powder used in the UHPC mixtures, which suggests a moderately rigorous standardization relative to the proportion of this component.

Regarding flexural strength, the box shows a reduced range, representing higher uniformity in the proportions of quartz powder used in UHPC mixtures, which indicates a more rigorous standardization relative to the consumption of this material.

Regarding elastic modulus, the box range increases compared to the previous case, suggesting higher variability between the quantities of quartz powder used in the UHPC mixtures. This represents a more flexible standardization relative to the consumption of this constituent material.

### 3.5. Influence of Mineral Additions from Waste on the UHPC Mixture

**Figure 11:**
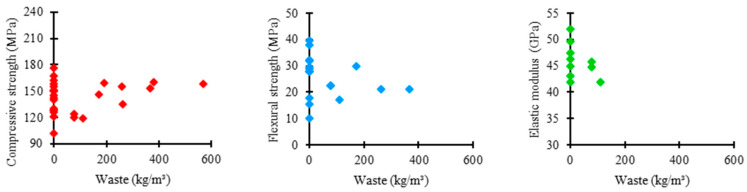
Proportion of mineral additions from waste × mechanical properties.

Figure 11 shows the relationship between the consumption of mineral additions derived from waste in the UHPC mixtures of the analyzed data and the respective material’s mechanical properties. As presented by most authors, waste was not used in concrete mixtures. However, additions were limited to 400 kg/m^3^ in studies that used the material. In this range, the compressive strength varied between 120 and 180 MPa, flexural strength between 10 and 40 MPa, and elastic modulus between 40 and 50 GPa.

**Figure 12:**
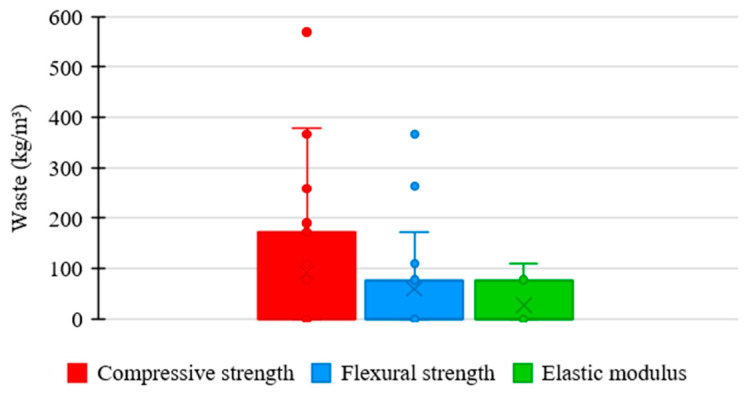
Tendency of mineral additions from waste consumption distribution × mechanical properties of UHPC.

Figure 12 shows the trends in the consumption of mineral additions derived from waste in the UHPC mixtures in the examined research regarding compressive strength, flexural strength, and elastic modulus.

The mean trend in the amount of waste among the ultra-high-performance concrete mixtures was 0 kg/m^3^ in the three analyzed mechanical properties. It shows that mineral additions from waste are not materials commonly used in research regarding the properties of UHPC.

However, the box range for compressive strength is higher compared to the others, indicating higher variability between the amounts of waste used in UHPC mixtures, which suggests a more flexible standardization relative to the proportion of this component.

Regarding flexural strength, the box shows a reduced range, representing higher uniformity in the proportions of waste used in UHPC mixtures, which indicates a more rigorous standardization relative to the consumption of this material.

Regarding elastic modulus, the box range shows the same size as the previous case, suggesting higher uniformity between the amounts of waste used in UHPC mixtures, which represents a rigorous standardization relative to the consumption of this constituent material.

### 3.6. Influence of Water on the UHPC Mixture

**Figure 13:**
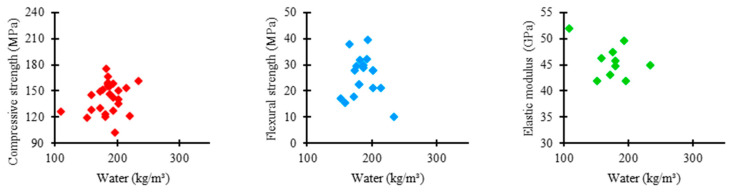
Proportion of water × mechanical properties.

Figure 13 shows the relationship between the amount of water used in the UHPC mixtures in the consulted studies and the corresponding mechanical properties. As presented by most authors, water consumption tends to converge between 150 and 250 kg/m^3^. In this range, the compressive strength varied between 120 and 180 MPa, flexural strength between 10 and 40 MPa, and elastic modulus between 40 and 50 GPa.

**Figure 14:**
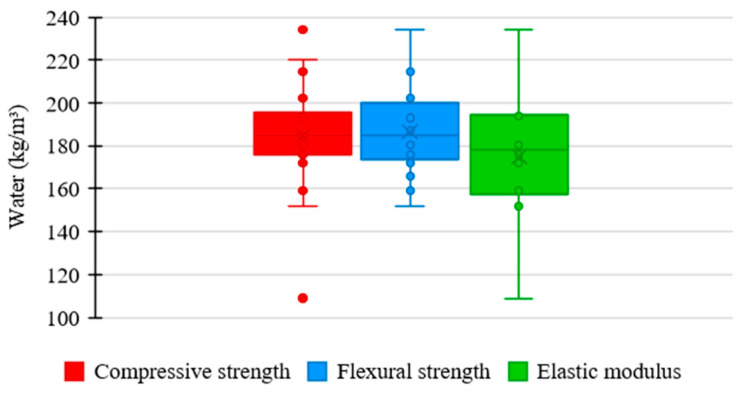
Tendency of water consumption distribution × mechanical properties of UHPC.

Figure 14 shows the distribution trends of water consumption in UHPC mixtures in the analyzed studies regarding compressive strength, flexural strength, and elastic modulus.

Unlike fine materials, the relationship between water content and concrete’s mechanical properties can be inversely proportional. Water incorporation aims to guarantee concrete workability, and therefore, the content is maintained at minimum values so that the material does not compromise the strength gain.

Regarding compressive strength, the mean trend in the amount of water among the concrete mixtures was 185 kg/m^3^, the highest value recorded among the analyzed properties. The increase in water content can be justified by the need to guarantee more significant homogeneity and compactness in UHPC, which are fundamental factors for the material to efficiently support the application of compressive loads. Another important factor is the reduced width of the box, which reveals a significant uniformity between the proportions of water used in the mixtures, establishing a rigorous standardization relative to the quantity of this material.

Regarding flexural strength, the mean trend in water consumption among concrete mixtures was 185 kg/m^3^. Similar to the previous case, the efficiency of UHPC in terms of flexural strength can be achieved by ensuring the homogeneity and compactness of the material, which can be obtained by increasing the water content. However, the box shows an increase in its range, indicating a moderate uniformity between the amounts of water in the mixtures, which reveals a slightly strict standardization relative to the consumption of this constituent.

The mean trend in the proportion of water between the concrete mixtures for the elastic modulus was 178 kg/m^3^, the lowest value recorded among the examined properties. It demonstrates that, unlike previous cases, the effectiveness of UHPC in terms of elastic modulus is not related to homogeneity or compactness but to the stiffness of the cementitious matrix. The material becomes more rigid by reducing water content and supporting higher loads without excessive deformation. Moreover, the high box range shows higher variability between the water consumption incorporated in the mixtures, suggesting a more flexible standardization relative to the quantity of this component.

### 3.7. Influence of Superplasticizer on the UHPC Mixture

**Figure 15:**
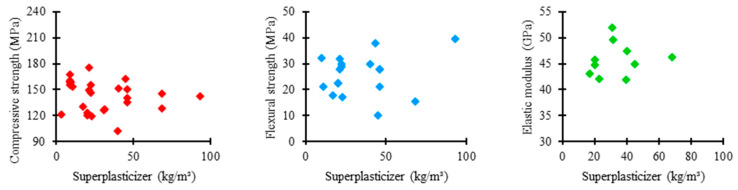
Proportion of superplasticizer × mechanical properties.

Figure 15 shows the relationship between the amount of superplasticizer used in the UHPC mixtures in the analyzed studies and the corresponding mechanical properties. As presented by most authors, the consumption of superplasticizers tends to be limited to 80 kg/m^3^. In this range, the compressive strength varied between 120 and 180 MPa, flexural strength between 10 and 40 MPa, and elastic modulus between 40 and 50 GPa.

**Figure 16:**
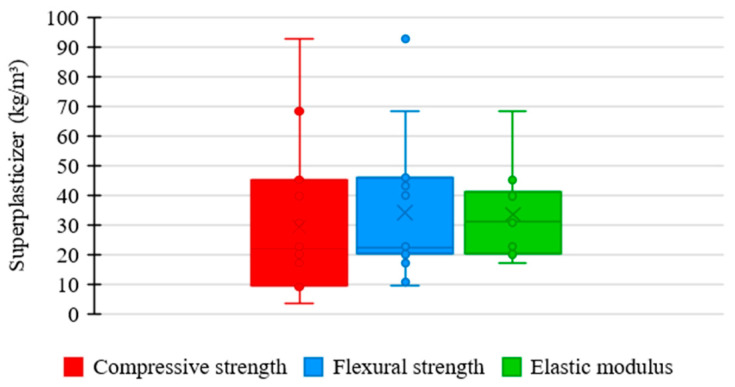
Tendency of superplasticizer consumption distribution × mechanical properties of UHPC.

Figure 16 shows the distribution trends of superplasticizer consumption in UHPC mixtures in the consulted studies regarding compressive strength, flexural strength, and elastic modulus.

Regarding compressive strength, the mean trend in the amount of superplasticizer among the concrete mixtures was 22 kg/m^3^, the lowest value recorded among the analyzed properties. It shows that the superplasticizer content considerably influences the compressive strength of UHPC, meaning that excessive consumption of the material is not necessary to achieve satisfactory results. However, the box has a high range, suggesting a significant dispersion between the amounts of superplasticizer used in the mixtures, which indicates a more flexible standardization relative to the proportion of this material.

Regarding flexural strength, the mean trend in superplasticizer consumption among concrete mixtures was 22 kg/m^3^, a value similar to that described in the previous case. It establishes that the influence of superplasticizer content on flexural strength is similar to that of compressive strength, not requiring a high amount of material to achieve satisfactory performance. Furthermore, the median range of the box demonstrates a moderate uniformity between the proportions of superplasticizers in the mixtures, revealing a slightly rigorous standardization relative to the consumption of this component.

Regarding elastic modulus, the mean trend in the proportion of superplasticizer among the concrete mixtures was 31 kg/m^3^, the highest value recorded among the examined properties. It indicates that the superplasticizer content does not significantly influence the elastic modulus of UHPC, requiring a higher consumption of the material to achieve satisfactory results. However, the box has a reduced range, suggesting considerable uniformity between the amounts of superplasticizer used in the mixtures, which suggests a rigorous standardization relative to the proportion of this constituent.

### 3.8. UHPC Mixture Proposal

Table 3 shows the mean values of the constituent materials of UHPC determined through the best results of compressive strength (157 MPa), flexural tensile strength (30 MPa), and elastic modulus (47 GPa). The results obtained for the dosage and mechanical properties of UHPC are in line with those proposed in the literature [59,60], which characterize ultra-high-performance concrete by its high cement consumption, predominance of fine aggregates, and low water-to-binder ratio, which suggests the existence of a pattern of values relative to the UHPC trace.

The water-to-binder ratio remained close to the limits between 0.10 and 0.20 despite small divergences between the mixtures, as recommended in the literature [20]. Furthermore, the amount of water remained constant among the three proposed formulations, suggesting that variations in the mechanical properties of UHPC are predominantly influenced by changes in the proportions of fine materials such as cement, fine aggregate, and silica fume.

### 3.9. Relationship between Theoretical and Experimental Results

#### 3.9.1. Compressive Strength

**Figure 17:**
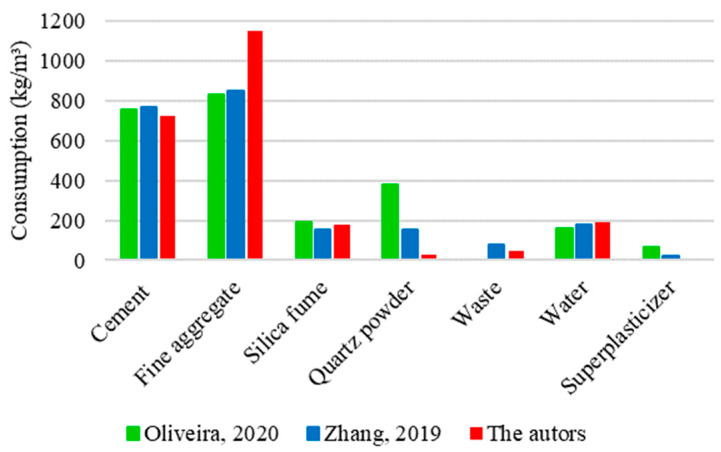
UHPC mixtures for compressive strength based on Oliveira [16] and Zhang [34].

Figure 17 shows a comparative analysis between three mixtures used to determine the compressive strength of UHPC. One of these mixtures represents the theoretical results obtained in this research, while the others correspond to experimental studies carried out by Oliveira [16] and Zhang [34].

The graphs show notable similarities in the proportions of cement, silica fume, and water between the analyzed UHPC mixtures. On average, the quantities were 800 kg/m^3^ of cement, 200 kg/m^3^ of silica fume, and 200 kg/m^3^ of water. On the other hand, significant divergences could be identified in the proportions of fine aggregate and quartz powder. The amount of fine aggregate for the mixture prepared in this study was established at 1150 kg/m^3^, representing an increase of 35% relative to data from Oliveira [16] and Zhang [34], who used 833 and 848 kg/m^3^, respectively. Oliveira [16] added 380 kg/m^3^ of quartz powder to the mixture, Zhang [34] used only 40% of this value (154 kg/m^3^), and 7% (25 kg/m^3^) was used in this research.

**Figure 18:**
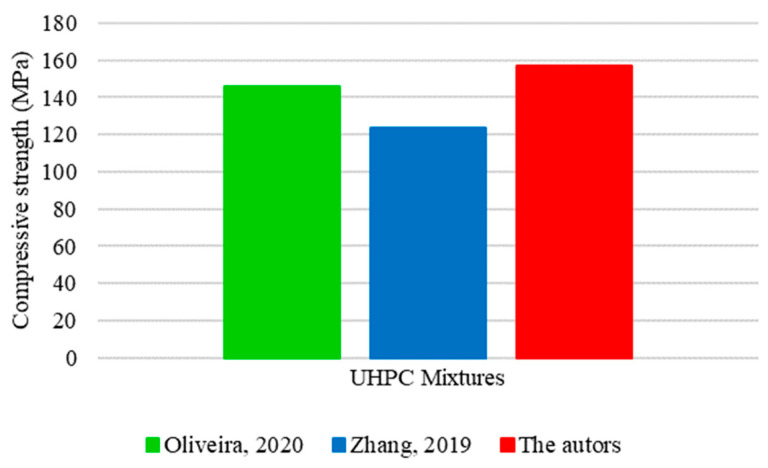
Values obtained for the compressive strength of UHPC based on Oliveira [16] and Zhang [34].

Discrepancies recorded between mixtures generated variations in the compressive strength values, as shown in Figure 18.

The mixture formulated in this study presented the highest compressive strength value among the analyses, reaching 157 MPa. The superior performance of concrete can be attributed to the significantly higher consumption of fine aggregate compared to the other mixtures. Despite having the lowest proportion of fine aggregate among the studies, Oliveira [16] compensated for this limitation through a substantial consumption of quartz powder, resulting in an intermediate strength of 146 MPa. In contrast, the concrete developed by Zhang [34] had the lowest compressive strength among the mixtures, reaching only 124 MPa. This drop in resistance can be justified by the intermediate amount of fine aggregate and quartz powder used in the composition of UHPC. The absence of a substantial consumption of fine materials can hinder the performance of concrete, especially regarding strength gains.

The final results corroborate previous studies on the relevance of particle packing on concrete performance [61,62]. The efficiency and compactness in the arrangement of particles in the cement matrix can optimize the use of constituent materials, improving cohesion and interaction between them. It results in the formation of a dense matrix, contributing to a significant gain in compressive strength.

#### 3.9.2. Flexural Tensile Strength

**Figure 19:**
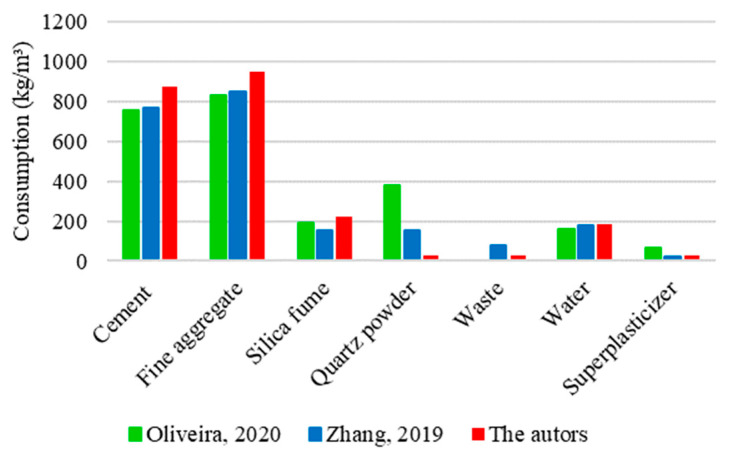
UHPC mixture for flexural tensile strength based on Oliveira [16] and Zhang [34].

Figure 19 shows a comparative analysis between three mixtures used to determine the flexural strength of UHPC. One of these mixtures corresponds to the results obtained in this study, while the others refer to the research developed experimentally by Oliveira [16] and Zhang [34].

The graphs reveal notable similarities regarding the amounts of silica fume and water incorporated into the analyzed UHPC mixtures. On average, consumption was 200 kg/m^3^ of silica fume and 200 kg/m^3^ of water. However, discrepancies could be observed in the cement, fine aggregate, and quartz powder consumptions. The cement consumption for the mixture prepared in this research was determined to be 875 kg/m^3^, a value 15% higher than the quantity used by Oliveira [16] (757 kg/m^3^) and 13% higher than that used by Zhang [34] (771 kg/m^3^). The proportion of fine aggregate was established to be 950 kg/m^3^ in this study, which corresponds to an increase of 14% compared to the study by Oliveira [16] and 12% by Zhang [34], who used 833 and 848 kg/m^3^ of fine aggregate in the mixtures, respectively. Regarding quartz powder consumption, Oliveira [16] used 380 kg/m^3^ in the concrete mixture, Zhang [34] used only 40% of this value (154 kg/m^3^), and 7% (25 kg/m^3^) was used in this research.

**Figure 20:**
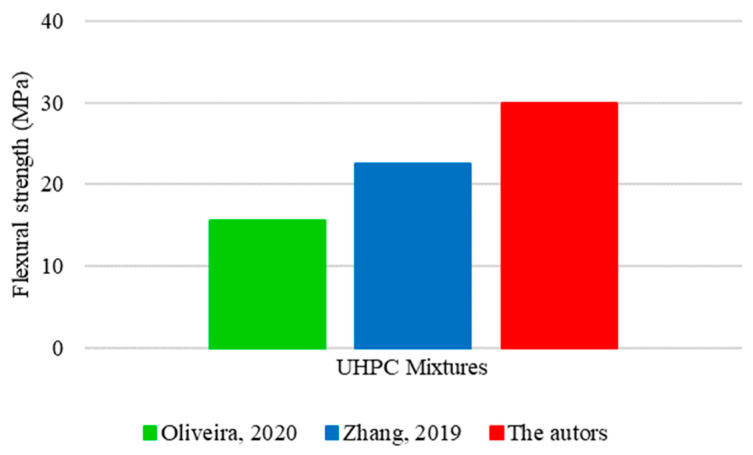
Values obtained for the flexural tensile strength of UHPC based on Oliveira [16] and Zhang [34].

The variations identified between the mixture compositions had a variation in the flexural tensile strength of UHPC, as shown in Figure 20.

The mixture proposed in this study had the highest flexural strength value, reaching 30 MPa. The excellence shown in concrete performance can be justified by the significantly higher consumption of cement and fine aggregate compared to other mixtures. Zhang [34] used intermediate amounts of cement, fine aggregate, and quartz powder in the mixture and obtained an intermediate strength of 23 MPa. Despite compensating for the disparity in the proportions of cement and fine aggregate with a significant quartz powder consumption, Oliveira [16] recorded the lowest flexural strength, reaching only 16 MPa. It suggests that, unlike the behavior recorded for compressive strength, in which quartz powder exerted a notable influence, its effect on flexural strength is less significant.

Given that the conditions relating to steel fibers were the same for the three analyzed mixtures (straight fibers at a content of 2% of the volume), the distinction in flexural strength results is mainly due to variations in cement and fine aggregate proportions. Although quartz powder is relevant in the context of UHPC, it has not been shown to have such a significant impact on the gain in flexural strength.

#### 3.9.3. Elastic Modulus

**Figure 21:**
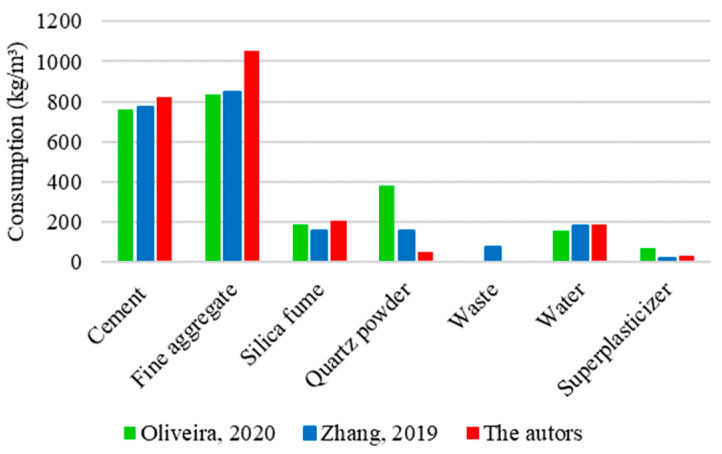
UHPC mixture for elastic modulus based on Oliveira [16] and Zhang [34].

Figure 21 shows a comparative analysis between three mixtures used to determine the elastic modulus of UHPC. One of these mixtures reflects the theoretical results of this research, while the others represent experimental studies conducted by Oliveira [16] and Zhang [34]. Figure 21 indicates significant similarities in the proportions of cement, silica fume, and water between the analyzed UHPC mixtures. On average, the quantities used were 800 kg/m^3^ of cement, 200 kg/m^3^ of silica fume, and 200 kg/m^3^ of water. However, divergences could be identified in the proportions of fine aggregate and quartz powder. A fine aggregate consumption of 1050 kg/m^2^ was determined for the mixture prepared in this study, corresponding to an increase of 26% relative to the research by Oliveira [16] and 24% relative to that of Zhang [34], which used 833 and 848 kg/m^3^ of material, respectively. As for quartz powder, Oliveira [16] added 380 kg/m^3^, Zhang [34] used only 40% of this value (154 kg/m^3^), and 13% (50 kg/m^3^) was used in this study.

**Figure 22:**
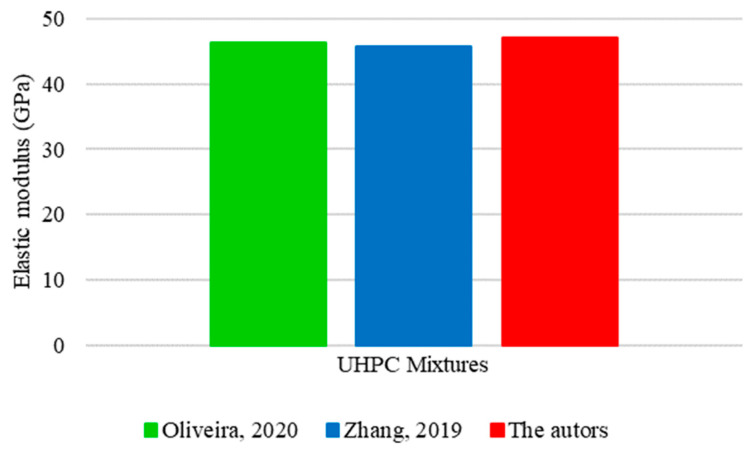
Values obtained for the elastic modulus of UHPC based on Oliveira [16] and Zhang [34].

The three analyzed mixtures generated no significant impacts on the elastic modulus of UHPC despite the discrepancies identified between them, as shown in Figure 22.

Even with a considerably higher consumption of fine aggregate compared to the other mixtures, this study obtained an elastic modulus of 47 GPa, corresponding to an increase of 2% relative to the research by Oliveira [16] and Zhang [34], who recorded values close to 46 GPa.

The final results corroborate previous studies [63], which found that the elastic modulus of ultra-high-performance concrete is considerably influenced by the reduction in the water-to-binder ratio and the addition of coarse aggregate. Given that the analyzed mixtures had similar water-to-binder ratios and included no coarse aggregate in their composition, it is consistent that the modulus of elasticity remains similar despite the variations identified between compositions.

### 3.10. Relationship between the Mechanical Properties of UHPC

**Figure 23:**
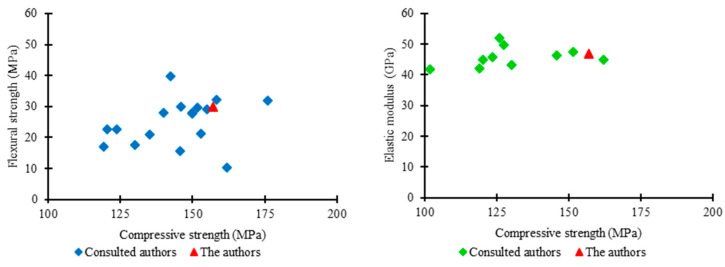
Relationships obtained for the mechanical properties of UHPC.

Figure 23 graphically shows the relationships between compressive strength, flexural tensile strength, and elastic modulus of UHPC. The results show that the relationship between compressive strength and flexural strength has a behavior contrary to that presented by the relationship between compressive strength and elastic modulus, although both are characterized as non-deterministic.

The relationship between the compressive strength and flexural strength of UHPC reveals that, for a compressive strength of 150 MPa, the flexural strength can vary between 10 and 40 MPa. This indicates that the differences between the components of each author’s UHPC mixtures significantly impact the flexural tensile properties of ultra-high-performance concrete.

Regarding the relationship between the compressive strength and the elastic modulus of UHPC, the elastic modulus remains consistently between 40 and 50 GPa for the compressive strength varying between 100 and 175 MPa. This behavior shows that variations in the proportions of UHPC components do not generate notable impacts on the elastic modulus, as long as the water-to-binder ratio is maintained at similar rates, as well as the proportions of coarse aggregate.

## 4. Conclusions

The following conclusions can be drawn considering the comprehensive review of the literature and the results obtained in this study:

- The theoretical mixtures developed in this study based on each mechanical property demonstrated compliance with the standards identified in the literature review for ultra-high-performance concrete, which characterize the material by a significant consumption of cement, high presence of fine aggregates, and low water-to-binder ratio.
- The mixtures prepared in this study based on each mechanical property showed significant similarities despite small variations, suggesting a standardization in the composition of UHPC.
- The small variations seen in the mixes examined in this study for each mechanical attribute confirm that fine materials are the primary factors enhancing the strength of UHPC.
- The water-to-binder ratio remained constant between the three mixtures prepared in this research for each mechanical property despite the small variations in the proportions of fine materials, proving the importance of the role played by water relative to the workability of concrete.
- The theoretical mixtures developed in this study performed superiorly to the experimental mixtures proposed by other authors in terms of compressive strength, flexural tensile strength, and elastic modulus.
- Fine aggregate plays a significant role in gaining compressive and flexural strength of UHPC. On the other hand, quartz powder has a higher influence on compressive strength than on flexural strength.
- Elastic modulus is not influenced by the proportion of constituent materials of UHPC, as there is no coarse aggregate, which is considered the main factor determining this property.
- The relationship between the mechanical properties of UHPC exhibits non-deterministic behavior. Flexural strength can vary for the same compressive strength value. However, elastic modulus remains relatively constant, regardless of variations in compressive strength values.

These conclusions make it evident that particle packing is the main factor in ensuring the high performance of UHPC in terms of strength and durability. However, to ensure the optimal packing of particles, the future recommendation of this study is that the proposed reference mixture for the UHPC be experimentally studied to verify the influence of local materials on their mechanical properties. This includes the availability of materials, granulometry, and mineralogical composition. Furthermore, it is recommended that alternative aggregates be explored in producing UHPC, such as rice husk ash and recycled concrete aggregate, from a sustainability perspective. Laboratory tests are essential for evaluating the particle distribution, mixture compactness, and material microstructure. By analyzing these aspects, it becomes possible to verify how the particles of the constituent materials are organized and interact in the mixture.

However, the theoretical mixtures developed in this work exhibited characteristics and performances that make them suitable for assisting in the initial stages of UHPC mix design in experimental programs.

Finally, the work offers an in-depth insight into the complexity presented by the interactions of the constituent materials and the mechanical properties of UHPC, providing relevant information for the development and optimization of this high-performance material in various engineering applications.

## Figures and Tables

**Table 1:** Criteria for including and excluding data in the collection process.

Inclusion Criteria	Exclusion Criteria
Experimental research paper	Analytical paper or numerical investigation
Mechanical properties of compressive and flexural strength, elastic modulus	Other types of mechanical properties (shear, fatigue, impact)
Moist curing	Heat curing
Age of rupture of the specimens equal to 28 days	Age of rupture of the specimens other than 28 days
Reinforcement with steel fibers	Reinforcement with fibers of materials other than steel
Fibers with a length of 13–20 mm	Fiber with a length less than 13 mm or greater than 20 mm
Straight fiber	Fiber other than straight (corrugated, with anchoring at the ends)
Fiber content equal to 2%	Fiber content other than 2%
Consumption of materials expressed in mass per volume (kg/m^3^)	Material consumption expressed in unit trace

**Table 2:** Mechanical properties of concrete in selected studies.

Reference	Compressive Strength (MPa)	Flexural Strength (MPa)	Elastic Modulus (GPa)
Feng et al. [35]	127.61	-	49.70
Gesoglu et al. [21]	162.00	10.15	45.00
Jiang et al. [56]	121.20	-	-
Kang et al. [22]	167.00	-	-
	159.00	-	-
	160.00	-	-
	158.00	-	-
	155.00	-	-
Li et al. [32]	128.90	30.00	51.50
Li and Rangaraju [23]	158.20	32.10	-
Máca et al. [24]	151.70	29.70	47.50
Meng and Khayat [25]	153.00	21.30	-
Oliveira [16]	145.70	15.60	46.22
Poncetti et al. [36]	102.00	-	41.85
Prado [7]	128.60	-	-
Ren et al. [37]	119.20	17.00	42.00
Tafraoui et al. [26]	155.00	29.00	-
	146.00	30.00	-
Valikhani et al. [38]	126.00	-	52.00
Wu et al. [27]	149.84	27.80	-
Wu et al. [33]	-	37.90	-
Wu et al. [28]	142.20	39.70	-
You et al. [29]	130.13	17.69	43.13
Yu et al. [30]	150.00	28.00	-
	135.00	21.00	-
	140.00	28.00	-
Zhang et al. [34]	120.40	22.60	44.80
	123.60	22.50	45.70
Zhong et al. [31]	176.00	32.00	-
Khaled et al. [57]	160.00		57.00
Guo et al. [58]	135.00	24.00	

**Table 3:** Mixtures obtained for UHPC as a function of mechanical properties.

Material (kg/m^3^)	Influence Mechanical Property		
	Compressive Strength (157 MPa)	Flexural Tensile Strength (30 MPa)	Elastic Modulus (47 GPa)
Cement	725	875	825
Fine Aggregate	1150	950	1050
Silica Fume	180	225	210
Quartz Powder	25	25	50
Mineral Additions from Waste	50	25	10
Water	190	185	190
Superplasticizer	5	25	35
Water-to-binder ratio	0.21	0.17	0.18

## Data Availability

Data are contained within the article.
